# Age modifies the relationship between ultra-processed food intake and hyperuricemia: findings from NHANES 1999–2018

**DOI:** 10.1186/s12889-026-26301-y

**Published:** 2026-01-21

**Authors:** Yue Yang, Lianchi Li, Hongxia Pan, Jianmei Zhang

**Affiliations:** 1 Rehabilitation Medicine Center and Institute of Rehabilitation Medicine, West China Hospital, Sichuan University, No. 37, Guoxue Road, Wuhou District, Chengdu, Sichuan Province 610041 China; 2 Department of Critical Care Medicine, West China Hospital, Sichuan University, Chengdu, China; 3 West China School of Nursing, Sichuan University, Chengdu, China

## Abstract

**Background:**

Hyperuricemia is a growing public health concern linked to gout, cardiovascular diseases, and metabolic syndrome. Ultra-processed foods (UPFs), characterized by industrial formulation with additives and minimal whole food content, have been hypothesized to be linked to uric acid overproduction through high levels of sugars, unhealthy fats, and purine precursors. This study aimed to investigate the association between UPF consumption and hyperuricemia across different age groups, hypothesizing that higher UPF intake is associated with higher odds of hyperuricemia, particularly in individuals under 60 years.

**Methods:**

Data from 43,713 adults aged ≥ 20 years from the National Health and Nutrition Examination Survey (NHANES) 1999–2018 were analyzed. Logistic regression models adjusted for sociodemographic, clinical, and lifestyle factors were used to assess the association between UPF ratio and hyperuricemia. Generalized additive models and stratified analyses were employed to evaluate non-linear relationships and potential interactions.

**Results:**

In fully adjusted models, each unit increase in UPF ratio was associated with 31% higher odds of hyperuricemia in the total population (OR 1.31, 95% CI 1.15–1.48, *P* < 0.001). This association was strongest among middle-aged adults (45–59 years) (OR 1.49, 95% CI 1.15–1.92, *P* < 0.01), followed by young adults (< 45 years) (OR 1.35, 95% CI 1.10–1.66, *P* < 0.01), while no significant association was observed among older adults (≥ 60 years). Significant interactions modifying the UPF-hyperuricemia association were observed for race/ethnicity, drinking status, and diabetes in young adults, and for gender, race/ethnicity, and drinking status in older adults.

**Conclusions:**

UPF consumption is significantly associated with higher odds of hyperuricemia in adults under 60 years, with the strongest association observed in middle-aged adults. Age substantially modifies this relationship, with no significant association in adults aged ≥ 60 years. These findings suggest that age-specific dietary recommendations targeting UPF reduction may be relevant for hyperuricemia management, particularly in younger and middle-aged populations.

**Supplementary Information:**

The online version contains supplementary material available at 10.1186/s12889-026-26301-y.

## Introduction

Hyperuricemia, characterized by elevated serum uric acid levels, is a significant public health concern due to its links with gout, cardiovascular diseases, and metabolic syndrome [1–3]. The global prevalence of hyperuricemia is rising, coinciding with dietary shifts toward energy-dense, nutrient-poor foods, particularly ultra-processed foods (UPFs) [1, 4–6]. UPFs are industrially formulated products containing additives and minimal whole food components. They are typically high in sugars, unhealthy fats, and purine precursors—components hypothesized to promote uric acid overproduction [4, 7]. Given the widespread consumption of UPFs across diverse populations, understanding their relationship with hyperuricemia has become increasingly important [8, 9]. 

Growing evidence supports an association between UPF consumption and hyperuricemia, with several studies documenting this relationship in different populations [10–12]. However, the potential for age to modify this association remains largely unexplored. Age is a critical factor in the epidemiology of hyperuricemia. Metabolic and renal functions vary across the lifespan, potentially modifying the relationship between dietary factors and serum uric acid levels [13, 14]. Younger adults typically have higher metabolic rates and greater UPF consumption. This population may exhibit stronger associations with hyperuricemia, possibly due to differences in uric acid production and renal clearance [15, 16]. Conversely, older adults often experience age-related declines in kidney function, which may independently be associated with elevated serum uric acid levels, potentially attenuating dietary effects [17, 18]. Additionally, UPF consumption has been related to obesity and type 2 diabetes [19, 20], both established conditions associated with hyperuricemia, yet few studies have examined whether these dietary relationships vary by age.

The primary objective of this study is to evaluate the association between the UPF consumption ratio and hyperuricemia across age groups using data from the National Health and Nutrition Examination Survey (NHANES) 1999–2018, hypothesizing that higher UPF intake is associated with higher odds of hyperuricemia, particularly in individuals aged < 60 years. Using logistic regression, generalized additive models, and stratified analyses, we aim to elucidate both linear and nonlinear relationships and potential age interactions. This comprehensive approach seeks to address the gap in understanding age-specific dietary associations with hyperuricemia and inform targeted public health strategies.

## Methods

### Study design and participants

The NHANES, conducted by the Centers for Disease Control and Prevention (CDC), served as the primary data source for this study, providing comprehensive data on the health and nutritional status of the U.S. population. NHANES employs a complex, multistage probability sampling design to obtain a nationally representative sample of non-institutionalized civilians. Briefly, the sampling design involves four stages: (1) selection of primary sampling units (counties or groups of counties), (2) selection of segments within primary sampling units, (3) selection of households within segments, and (4) selection of individuals within households. Certain population subgroups, including non-Hispanic Black individuals, Hispanic individuals, and older adults, were oversampled to ensure adequate sample sizes for subgroup analyses. The survey integrates structured interviews, physical examinations, and laboratory tests, collecting data on demographics, dietary intake, and biomarkers essential for examining associations between dietary factors and health outcomes, such as hyperuricemia. This study utilized data from NHANES cycles spanning 1999 to 2018, combining multiple cycles to increase sample size and enhance statistical power for cross-sectional analyses. The standardized protocols of NHANES ensure consistency in data collection across cycles. This makes NHANES a robust resource for investigating the relationship between UPF consumption and hyperuricemia across diverse age groups.

Participants were selected based on specific inclusion and exclusion criteria to ensure the study population aligned with the research objectives. Inclusion criteria required participants to be aged ≥ 20 years, focusing on adults for whom the association between dietary factors and hyperuricemia may be more pronounced given dietary and metabolic factors. Exclusion criteria were applied to minimize bias and ensure data completeness. Pregnant individuals were excluded, as pregnancy is associated with changes in uric acid metabolism and dietary patterns. Additionally, participants with missing data on UPF intake, the primary exposure variable, were excluded to ensure accuracy in assessing dietary patterns. Similarly, those lacking serum uric acid measurements were excluded to ensure reliable evaluation of the outcome variable, hyperuricemia.

### Assessment of food consumption

Dietary intake data were collected using the 24-hour dietary recall method via the Automated Multiple-Pass Method, a standardized approach implemented in the NHANES. Participants completed two 24-hour dietary recalls: the first was conducted in person at the Mobile Examination Center, and the second was administered via telephone 3 to 10 days later. This method captures detailed information on all foods and beverages consumed in the preceding 24 h, including portion sizes and preparation methods. Foods were classified as ultra-processed using the NOVA food classification system, which categorizes foods based on the extent and purpose of industrial processing [21, 22]. UPFs were defined as products containing multiple ingredients, including additives, and minimal whole food components, such as sugar-sweetened beverages, packaged snacks, and ready-to-eat meals. Details on the UPFs included in this study are provided in Table S1. The UPF consumption ratio, calculated as the proportion of total energy intake derived from UPFs and expressed as a percentage of daily energy intake, served as the primary exposure variable. This ratio enabled a standardized assessment of the association between UPF consumption and hyperuricemia across the study population.

### Definition of hyperuricemia

Hyperuricemia was defined based on serum uric acid concentrations measured in the NHANES participants. Serum uric acid levels were quantified using standardized enzymatic methods, involving uricase-mediated oxidation of uric acid to produce allantoin and hydrogen peroxide, followed by colorimetric detection. Hyperuricemia was classified using gender-specific thresholds to account for physiological differences: serum uric acid levels > 7.0 mg/dL for men and > 6.0 mg/dL for women were considered indicative of hyperuricemia. These thresholds are widely accepted in clinical and epidemiological research for identifying elevated uric acid levels associated with higher prevalence of gout and other metabolic disorders [14, 23]. Participants meeting these criteria were categorized as having hyperuricemia, which served as the primary outcome variable for evaluating its association with UPF consumption across age groups in the study population.

### Covariates

To adjust for potential confounders in the association between UPF consumption ratio and hyperuricemia, several covariates were included in the analysis, sourced from the NHANES dataset. Sociodemographic characteristics and lifestyle factors were obtained through standardized computer-assisted personal interviews. Clinical and laboratory measurements were collected during health examinations at the mobile examination center using calibrated equipment and validated protocols. Demographic factors included gender (male or female) and race/ethnicity (non-Hispanic White, non-Hispanic Black, Mexican American, or other race, including multiracial and other Hispanic). Socioeconomic variables comprised the poverty income ratio (PIR; categorized as low [< 1.3], medium [1.3–3.5], or high [> 3.5]) and education level (less than high school [e.g., <9th grade, 9th-11th grade, or 12th grade without diploma], high school graduate [including GED or equivalent], or more than high school [some college, associate’s degree, or college graduate]) [24]. Lifestyle factors included smoking status (never, former, or current) [25], alcohol consumption (never, former, or current [mild, moderate, or heavy]) [26], and physical activity, measured as metabolic equivalent tasks (METs/week; categorized as low [< 600], moderate [600–1200], or vigorous [≥ 1200]) [27, 28]. Health-related covariates included body mass index (BMI; kg/m²), calculated from measured weight and height. Anthropometric measurements were performed by trained health technologists at the mobile examination center following standardized protocols described in the NHANES Anthropometry Procedures Manual. Body weight was measured using a calibrated digital floor scale with participants wearing a standard examination gown. Standing height was measured using a fixed stadiometer, with participants standing erect, heels together, and head positioned in the Frankfort horizontal plane. Other health-related covariates included estimated glomerular filtration rate (eGFR; mL/min/1.73 m², calculated using the CKD-EPI equation) [29], and comorbidities, specifically diabetes (yes or no, defined as self-reported physician diagnosis, glycohemoglobin > 6.5%, fasting glucose ≥ 7.0 mmol/L, random glucose ≥ 11.1 mmol/L, or 2-hour oral glucose tolerance test glucose ≥ 11.1 mmol/L) [30], hypertension (yes or no, defined as mean blood pressure ≥ 140/90 mmHg, self-reported history, or antihypertensive medication use; mean blood pressure was calculated from available readings excluding the first measurement and any diastolic readings of zero) [31], and hyperlipidemia (yes or no, defined as triglycerides ≥ 150 mg/dL, total cholesterol ≥ 200 mg/dL, LDL cholesterol ≥ 130 mg/dL, HDL cholesterol < 40 mg/dL for men or < 50 mg/dL for women, or use of lipid-lowering medication) [32]. For the sensitivity analysis, urate-lowering therapy use was defined as self-reported use of allopurinol, febuxostat, or probenecid based on the NHANES prescription medication questionnaire.

### Statistical analysis

Statistical analyses were conducted to evaluate the association between the UPF consumption ratio and hyperuricemia, with an emphasis on age-stratified associations. Continuous variables were described as survey-weighted means (95% confidence interval (CI)), and categorical variables were presented as survey-weighted percentages (95% CI). Differences in baseline characteristics between hyperuricemia and non-hyperuricemia groups were assessed using survey-weighted linear regression for continuous variables and survey-weighted Chi-square tests for categorical variables. Logistic regression models were used to estimate odds ratios (ORs) and 95% CIs for hyperuricemia across UPF consumption ratio quartiles and as a continuous variable. Three models were applied: Model 1 was unadjusted; Model 2 adjusted for gender; and Model 3 further adjusted for race/ethnicity, poverty income ratio, education level, body mass index, smoking status, alcohol consumption, diabetes, hypertension, hyperlipidemia, physical activity (METs/week), and estimated glomerular filtration rate. Model diagnostics were conducted to assess the validity of logistic regression analyses. Multicollinearity among covariates was evaluated using generalized variance inflation factors (GVIF), with GVIF^(1/(2×Df)) < 2 considered acceptable. Model goodness-of-fit was assessed using the Hosmer-Lemeshow test. Model discrimination was evaluated using the C-statistic (area under the receiver operating characteristic curve, AUC), with values ≥ 0.7 indicating acceptable predictive performance. To quantify the potential impact of unmeasured confounding, E-values were calculated for significant associations, representing the minimum strength of association that an unmeasured confounder would need to have with both the exposure and outcome to fully explain the observed association, on the risk ratio scale. Generalized additive models with smooth curve fitting were used to explore potential nonlinear relationships between the UPF consumption ratio and hyperuricemia in the total population and by age group [33]. Participants were stratified into three age groups: young adults (< 45 years), middle-aged adults (45–59 years), and older adults (≥ 60 years), consistent with prior epidemiological studies on hyperuricemia and dietary factors [34, 35]. Interaction effects between the UPF consumption ratio and age were visualized using heatmaps and three-dimensional plots. Missing data for covariates were handled using multiple imputation by chained equations (MICE) with 5 imputed datasets. The plausibility of imputations was assessed using density plots comparing the distributions of observed and imputed values for covariates with missing data. As a sensitivity analysis to assess the robustness of the findings to the handling of missing data, a complete case analysis was also performed, restricting the analysis to participants with no missing data on any covariate included in the regression models, using the same logistic regression models with the UPF consumption ratio as a continuous variable. Additionally, a sensitivity analysis was conducted by further adjusting for urate-lowering therapy use to evaluate whether the observed associations were influenced by pharmacological treatment of hyperuricemia. All analyses incorporated NHANES survey weights to ensure nationally representative estimates, with a P-value < 0.05 considered statistically significant. All statistical analyses were performed using R version 4.4.0 (R Foundation for Statistical Computing, Vienna, Austria).

## Results

### Participant selection process

**Fig. 1:**
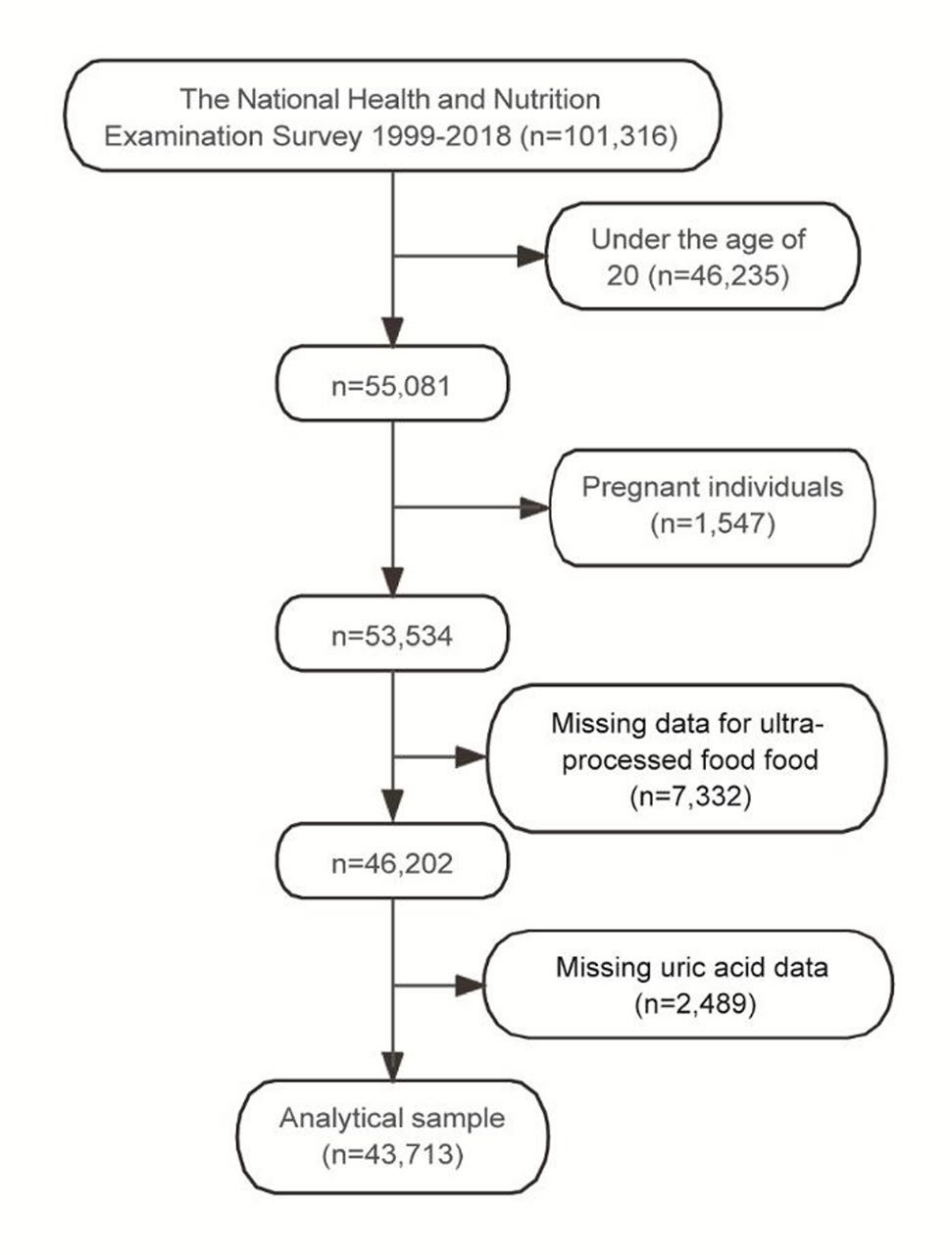
Flowchart of participant selection for the study

The participant selection process, illustrated in Fig. 1, began with an initial sample of 101,316 individuals from the NHANES cycles spanning 1999 to 2018. Participants were excluded based on predefined criteria: 46,235 individuals aged < 20 years were excluded, leaving 55,081 participants. Subsequently, 1547 pregnant individuals were excluded, reducing the sample to 53,534. Further exclusions included 7332 participants with missing UPF consumption data, resulting in 46,202 participants, and 2489 participants with missing serum uric acid data, yielding a final analytical sample of 43,713 participants.

### Baseline demographic characteristics

Tables 1 and 2 presents the baseline characteristics of the study population, stratified by age group and hyperuricemia status. Notable gender disparities were observed: men comprised a higher proportion of hyperuricemic individuals among younger (< 45 years; 72.57% vs. 47.49%) and middle-aged adults (45–59 years; 56.20% vs. 48.04%), whereas this pattern reversed in older adults (≥ 60 years; 40.94% vs. 46.63%) (all *P* < 0.0001). Race/ethnicity distributions varied significantly across age groups, with non-Hispanic Black individuals showing higher proportions among hyperuricemic participants in middle-aged (14.85% vs. 9.48%) and older adult groups (11.33% vs. 6.79%) (*P* < 0.0001). Socioeconomic factors were associated with hyperuricemia variably: poverty income ratio differed notably in middle-aged (*P* = 0.0147) and older adults (*P* < 0.0001), while educational attainment differed notably in younger (*P* = 0.0128) and middle-aged adults (*P* = 0.0229).

**Table 1:** Sociodemographic and lifestyle characteristics of the study population by age groups and hyperuricemia status

Variables	Young adults (< 45 years)				Middle-aged adults (45–59 years)				Older adults (≥ 60 years)			
	Total	Non-Hyperuricemia	Hyperuricemia	*P*-value	Total	Non-Hyperuricemia	Hyperuricemia	*P*-value	Total	Non-Hyperuricemia	Hyperuricemia	*P*-value
Age (years)	32.18 (31.98, 32.39)	32.19 (31.97, 32.40)	32.16 (31.76, 32.56)	0.9079	51.66 (51.54, 51.78)	51.58 (51.45, 51.72)	52.03 (51.75, 52.31)	0.0066	69.91 (69.71, 70.10)	69.62 (69.41, 69.82)	70.79 (70.43, 71.15)	< 0.0001
Gender (%)				< 0.0001				< 0.0001				< 0.0001
Male	51.15 (50.39, 51.90)	47.49 (46.66, 48.32)	72.57 (70.69, 74.38)		49.41 (48.42, 50.40)	48.04 (46.82, 49.26)	56.20 (53.58, 58.79)		45.24 (44.40, 46.08)	46.63 (45.65, 47.61)	40.94 (38.88, 43.03)	
Female	48.85 (48.10, 49.61)	52.51 (51.68, 53.34)	27.43 (25.62, 29.31)		50.59 (49.60, 51.58)	51.96 (50.74, 53.18)	43.80 (41.21, 46.42)		54.76 (53.92, 55.60)	53.37 (52.39, 54.35)	59.06 (56.97, 61.12)	
Race/ethnicity (%)				0.0001				< 0.0001				< 0.0001
Non-Hispanic White	62.71 (60.48, 64.88)	62.09 (59.82, 64.30)	66.36 (63.65, 68.96)		72.27 (70.07, 74.37)	72.86 (70.66, 74.95)	69.35 (66.06, 72.46)		80.56 (78.73, 82.27)	81.18 (79.34, 82.89)	78.66 (76.37, 80.78)	
Non-Hispanic Black	11.70 (10.55, 12.95)	11.84 (10.68, 13.10)	10.87 (9.49, 12.42)		10.38 (9.29, 11.58)	9.48 (8.45, 10.61)	14.85 (12.90, 17.02)		7.90 (6.97, 8.95)	6.79 (5.95, 7.74)	11.33 (9.89, 12.95)	
Mexican American	11.25 (9.99, 12.64)	11.57 (10.29, 12.98)	9.39 (7.86, 11.19)		6.26 (5.35, 7.31)	6.69 (5.72, 7.82)	4.12 (3.35, 5.06)		3.75 (3.05, 4.60)	4.04 (3.30, 4.94)	2.85 (2.20, 3.67)	
Others	14.34 (13.12, 15.66)	14.51 (13.24, 15.88)	13.38 (11.75, 15.20)		11.09 (9.98, 12.31)	10.97 (9.85, 12.21)	11.68 (9.83, 13.83)		7.79 (6.90, 8.78)	7.99 (7.08, 9.00)	7.17 (5.89, 8.70)	
PIR (%)				0.3701				0.0147				< 0.0001
Low	25.38 (24.14, 26.67)	25.62 (24.30, 26.98)	24.01 (22.12, 26.00)		15.91 (14.63, 17.26)	15.48 (14.11, 16.96)	17.99 (15.85, 20.34)		18.45 (17.19, 19.78)	17.65 (16.32, 19.07)	20.90 (19.21, 22.69)	
Medium	37.18 (35.92, 38.47)	37.10 (35.80, 38.41)	37.69 (35.13, 40.33)		29.65 (28.08, 31.28)	29.35 (27.68, 31.08)	31.15 (28.48, 33.95)		41.27 (39.75, 42.80)	40.48 (38.87, 42.11)	43.69 (41.33, 46.09)	
High	37.43 (35.78, 39.11)	37.29 (35.62, 38.99)	38.30 (35.31, 41.38)		54.44 (52.26, 56.60)	55.16 (52.91, 57.40)	50.86 (47.57, 54.14)		40.29 (38.22, 42.39)	41.87 (39.71, 44.06)	35.41 (32.58, 38.35)	
Education level (%)				0.0128				0.0229				0.0589
Less than high school	15.69 (14.75, 16.67)	15.90 (14.90, 16.95)	14.43 (12.99, 15.99)		14.56 (13.52, 15.66)	14.48 (13.38, 15.65)	14.95 (13.26, 16.81)		20.81 (19.46, 22.22)	20.34 (18.89, 21.88)	22.24 (20.46, 24.14)	
High school graduate	23.42 (22.27, 24.62)	23.00 (21.79, 24.24)	25.94 (23.67, 28.35)		23.40 (22.26, 24.59)	22.88 (21.62, 24.18)	26.02 (23.63, 28.56)		26.44 (25.31, 27.60)	26.22 (25.02, 27.46)	27.10 (25.08, 29.21)	
More than high school	60.89 (59.21, 62.54)	61.10 (59.38, 62.80)	59.63 (56.89, 62.31)		62.04 (60.25, 63.80)	62.65 (60.71, 64.54)	59.03 (56.14, 61.86)		52.75 (51.04, 54.46)	53.43 (51.57, 55.29)	50.66 (48.01, 53.30)	
Smoking (%)				0.0037				0.0003				< 0.0001
Never	57.80 (56.49, 59.09)	58.16 (56.83, 59.47)	55.68 (53.20, 58.14)		50.92 (49.49, 52.35)	51.46 (49.83, 53.08)	48.24 (45.69, 50.81)		48.28 (47.06, 49.50)	48.70 (47.37, 50.04)	46.95 (44.59, 49.32)	
Former	15.88 (15.03, 16.77)	15.43 (14.57, 16.34)	18.53 (16.59, 20.65)		26.84 (25.74, 27.97)	25.86 (24.61, 27.16)	31.68 (28.93, 34.57)		40.34 (39.24, 41.45)	39.10 (37.88, 40.34)	44.15 (41.98, 46.35)	
Now	26.32 (25.23, 27.44)	26.41 (25.25, 27.61)	25.78 (23.79, 27.88)		22.24 (20.99, 23.55)	22.68 (21.24, 24.18)	20.08 (18.02, 22.31)		11.38 (10.70, 12.11)	12.19 (11.39, 13.04)	8.90 (7.49, 10.54)	
Drinking (%)				0.1116				0.0640				0.0004
Never	9.92 (8.90, 11.03)	10.11 (9.14, 11.17)	8.77 (7.09, 10.81)		9.13 (8.29, 10.05)	9.15 (8.26, 10.12)	9.04 (7.54, 10.81)		15.51 (14.42, 16.67)	15.00 (13.85, 16.23)	17.10 (15.46, 18.87)	
Former	8.97 (8.29, 9.70)	9.05 (8.33, 9.83)	8.47 (7.30, 9.80)		16.02 (14.80, 17.32)	15.59 (14.35, 16.92)	18.15 (15.89, 20.65)		22.95 (21.79, 24.15)	22.32 (21.06, 23.64)	24.88 (22.96, 26.90)	
Current	81.11 (79.72, 82.43)	80.83 (79.47, 82.13)	82.76 (80.39, 84.91)		74.85 (73.23, 76.40)	75.26 (73.56, 76.88)	72.81 (70.01, 75.44)		61.54 (59.68, 63.37)	62.68 (60.70, 64.62)	58.02 (55.43, 60.58)	
METs/week (%)				0.2322				0.0232				0.1028
Low	33.60 (32.41, 34.81)	33.35 (32.13, 34.60)	35.06 (32.57, 37.63)		33.68 (32.27, 35.13)	32.99 (31.54, 34.48)	37.10 (34.01, 40.29)		34.80 (33.56, 36.06)	34.17 (32.71, 35.67)	36.72 (34.62, 38.87)	
Moderate	2.86 (2.56, 3.18)	2.93 (2.62, 3.29)	2.42 (1.80, 3.25)		3.07 (2.69, 3.49)	3.06 (2.66, 3.52)	3.10 (2.27, 4.23)		3.68 (3.26, 4.16)	3.63 (3.16, 4.17)	3.84 (3.05, 4.82)	
Vigorous	63.54 (62.28, 64.79)	63.71 (62.41, 65.00)	62.52 (59.87, 65.11)		63.25 (61.78, 64.70)	63.95 (62.42, 65.45)	59.80 (56.57, 62.94)		61.52 (60.25, 62.78)	62.19 (60.70, 63.67)	59.44 (57.23, 61.61)	

*Abbreviations: CI* Confidence interval, *PIR* Poverty income ratio, *METs* Metabolic equivalents

Notes: Data are presented as survey-weighted mean (95% CI) for continuous variables and survey-weighted percentage (95% CI) for categorical variables. P-values for continuous variables were calculated by survey-weighted linear regression; P-values for categorical variables were calculated by survey-weighted Chi-square test

**Table 2:** Anthropometric, clinical, and dietary characteristics of the study population by age groups and hyperuricemia status

Variables	Young adults (< 45 years)				Middle-aged adults (45–59 years)				Older adults (≥ 60 years)			
	Total	Non-Hyperuricemia	Hyperuricemia	*P*-value	Total	Non-Hyperuricemia	Hyperuricemia	*P*-value	Total	Non-Hyperuricemia	Hyperuricemia	*P*-value
Height (cm)	170.06 (169.88, 170.25)	169.41 (169.20, 169.61)	173.91 (173.51, 174.31)	< 0.0001	169.34 (169.08, 169.60)	169.16 (168.88, 169.44)	170.26 (169.65, 170.87)	0.0012	166.11 (165.86, 166.36)	166.36 (166.09, 166.63)	165.34 (164.91, 165.77)	< 0.0001
BMI (kg/m²)	28.29 (28.12, 28.46)	27.52 (27.35, 27.68)	32.85 (32.45, 33.24)	< 0.0001	29.44 (29.23, 29.66)	28.72 (28.51, 28.94)	33.01 (32.54, 33.49)	< 0.0001	28.97 (28.81, 29.12)	28.25 (28.09, 28.40)	31.18 (30.88, 31.47)	< 0.0001
Waist circumference (cm)	95.31 (94.89, 95.74)	93.21 (92.80, 93.62)	107.66 (106.70, 108.62)	< 0.0001	100.76 (100.24, 101.28)	98.99 (98.45, 99.53)	109.49 (108.51, 110.47)	< 0.0001	102.19 (101.79, 102.60)	100.56 (100.17, 100.96)	107.22 (106.46, 107.98)	< 0.0001
SBP (mmHg)	115.62 (115.30, 115.94)	114.81 (114.49, 115.12)	120.40 (119.67, 121.13)	< 0.0001	123.47 (122.99, 123.94)	122.73 (122.22, 123.24)	127.11 (126.06, 128.17)	< 0.0001	133.05 (132.51, 133.59)	132.83 (132.26, 133.40)	133.72 (132.75, 134.70)	0.0831
DBP (mmHg)	70.66 (70.33, 70.98)	70.04 (69.72, 70.36)	74.26 (73.61, 74.91)	< 0.0001	74.95 (74.59, 75.30)	74.60 (74.22, 74.98)	76.66 (75.98, 77.33)	< 0.0001	68.24 (67.86, 68.62)	68.65 (68.24, 69.06)	66.97 (66.38, 67.55)	< 0.0001
Uric acid (mg/dL)	5.31 (5.29, 5.34)	4.94 (4.92, 4.96)	7.54 (7.50, 7.58)	< 0.0001	5.41 (5.37, 5.44)	4.99 (4.96, 5.02)	7.45 (7.40, 7.51)	< 0.0001	5.65 (5.62, 5.68)	5.06 (5.03, 5.08)	7.49 (7.44, 7.53)	< 0.0001
eGFR (ml/min/1.73 m²)	107.10 (106.61, 107.59)	108.04 (107.54, 108.53)	101.61 (100.67, 102.55)	< 0.0001	90.75 (90.26, 91.23)	92.01 (91.54, 92.48)	84.49 (83.37, 85.61)	< 0.0001	72.92 (72.46, 73.39)	76.17 (75.68, 76.65)	62.93 (62.18, 63.69)	< 0.0001
Diabetes (%)				< 0.0001				< 0.0001				< 0.0001
No	95.54 (95.18, 95.87)	95.90 (95.51, 96.25)	93.46 (92.27, 94.47)		85.38 (84.41, 86.30)	86.33 (85.32, 87.28)	80.68 (78.25, 82.90)		73.87 (72.83, 74.87)	76.39 (75.34, 77.41)	66.08 (63.79, 68.30)	
Yes	4.46 (4.13, 4.82)	4.10 (3.75, 4.49)	6.54 (5.53, 7.73)		14.62 (13.70, 15.59)	13.67 (12.72, 14.68)	19.32 (17.10, 21.75)		26.13 (25.13, 27.17)	23.61 (22.59, 24.66)	33.92 (31.70, 36.21)	
Hypertension (%)				< 0.0001				< 0.0001				< 0.0001
No	82.58 (81.73, 83.39)	84.75 (83.92, 85.55)	69.83 (67.50, 72.06)		57.78 (56.30, 59.25)	61.97 (60.38, 63.55)	37.02 (33.97, 40.17)		32.23 (31.01, 33.46)	36.42 (35.04, 37.83)	19.28 (17.45, 21.25)	
Yes	17.42 (16.61, 18.27)	15.25 (14.45, 16.08)	30.17 (27.94, 32.50)		42.22 (40.75, 43.70)	38.03 (36.45, 39.62)	62.98 (59.83, 66.03)		67.77 (66.54, 68.99)	63.58 (62.17, 64.96)	80.72 (78.75, 82.55)	
Hyperlipidemia (%)				< 0.0001				< 0.0001				< 0.0001
No	40.54 (39.53, 41.56)	43.25 (42.16, 44.34)	24.67 (22.37, 27.12)		21.09 (19.89, 22.33)	22.73 (21.38, 24.14)	12.95 (11.04, 15.13)		14.77 (14.02, 15.57)	15.85 (14.96, 16.79)	11.44 (10.03, 13.02)	
Yes	59.46 (58.44, 60.47)	56.75 (55.66, 57.84)	75.33 (72.88, 77.63)		78.91 (77.67, 80.11)	77.27 (75.86, 78.62)	87.05 (84.87, 88.96)		85.23 (84.43, 85.98)	84.15 (83.21, 85.04)	88.56 (86.98, 89.97)	
Total energy intake (kcal)	2392.11 (2373.32, 2410.90)	2374.69 (2355.13, 2394.25)	2494.25 (2441.79, 2546.70)	< 0.0001	2210.53 (2186.98, 2234.07)	2214.15 (2187.80, 2240.50)	2192.58 (2136.22, 2248.94)	0.5040	1868.45 (1846.50, 1890.40)	1904.90 (1881.36, 1928.44)	1756.09 (1717.63, 1794.55)	< 0.0001
Ultra-processed food (kcal)	1040.03 (1024.39, 1055.67)	1023.45 (1007.81, 1039.08)	1137.26 (1099.05, 1175.47)	< 0.0001	851.14 (832.80, 869.48)	846.93 (826.92, 866.93)	871.99 (826.59, 917.39)	0.3229	621.24 (606.16, 636.32)	634.21 (617.21, 651.21)	581.25 (554.57, 607.93)	0.0007
Ultra-processed food ratio (%)	0.42 (0.42, 0.43)	0.42 (0.41, 0.42)	0.44 (0.43, 0.45)	< 0.0001	0.37 (0.37, 0.38)	0.37 (0.36, 0.38)	0.38 (0.37, 0.40)	0.0710	0.32 (0.32, 0.33)	0.32 (0.32, 0.33)	0.32 (0.31, 0.33)	0.4559

*Abbreviations: CI* Confidence interval, *BMI* Body mass index, *SBP* Systolic blood pressure, *DBP* Diastolic blood pressure, *eGFR* Estimated glomerular filtration rate

Notes: Data are presented as survey-weighted mean (95% CI) for continuous variables and survey-weighted percentage (95% CI) for categorical variables. P-values for continuous variables were calculated by survey-weighted linear regression; P-values for categorical variables were calculated by survey-weighted Chi-square test

Anthropometric and clinical parameters showed consistent patterns across age groups. Hyperuricemic individuals had significantly higher BMI in all age categories (*P* < 0.0001), with the largest difference in younger adults (32.85 vs. 27.52 kg/m²). Waist circumference was also significantly greater in hyperuricemic participants across all age groups (*P* < 0.0001). Blood pressure measurements differed significantly in younger and middle-aged adults (*P* < 0.0001 for systolic and diastolic), but diastolic blood pressure was significantly lower in hyperuricemic older adults (66.97 vs. 68.65 mmHg; *P* < 0.0001). eGFR was significantly lower in hyperuricemic participants across all age groups (*P* < 0.0001), with the most pronounced difference in older adults (62.93 vs. 76.17 mL/min/1.73 m²). Comorbidities, including diabetes, hypertension, and hyperlipidemia, were significantly more prevalent in hyperuricemic individuals across all age groups (all *P* < 0.0001).

Lifestyle and dietary patterns also varied by hyperuricemia status across age groups. Smoking status differed significantly in all age groups (younger adults: *P* = 0.0037; middle-aged adults: *P* = 0.0003; older adults: *P* < 0.0001), with a higher proportion of former smokers among hyperuricemic participants, particularly in older adults (44.15% vs. 39.10%). Alcohol consumption differed significantly only in older adults (*P* = 0.0004). Total energy intake was significantly higher in hyperuricemic younger adults (2494.25 vs. 2374.69 kcal; *P* < 0.0001) but significantly lower in hyperuricemic older adults (1756.09 vs. 1904.90 kcal; *P* < 0.0001). UPF consumption exhibited age-dependent patterns: hyperuricemic younger adults had significantly higher absolute UPF intake and proportion of total energy from UPFs (both *P* < 0.0001), whereas hyperuricemic older adults had significantly lower absolute UPF intake (581.25 vs. 634.21 kcal; *P* = 0.0007) but no significant difference in the proportion of total energy from UPFs (*P* = 0.4559).

### Association between UPF ratio and hyperuricemia stratified by age

Table 3 demonstrates a significant association between the UPF consumption ratio and hyperuricemia, with substantial variation by age group. In the fully adjusted model (Model 3), each unit increase in the UPF consumption ratio was associated with a 31% higher odds of hyperuricemia in the total population (OR, 1.31; 95% CI, 1.15–1.48; *P* < 0.0001). This association was strongest among middle-aged adults (45–59 years; OR, 1.49; 95% CI, 1.15–1.92; *P* = 0.002) and younger adults (< 45 years; OR, 1.35; 95% CI, 1.10–1.66; *P* = 0.004), but no significant association was observed in older adults (≥ 60 years; OR, 1.17; 95% CI, 0.95–1.43; *P* = 0.138). When analyzed by quartiles, individuals in the highest UPF consumption quartile (Q4) had significantly higher odds of hyperuricemia compared with those in the lowest quartile (Q1) in the total population (OR, 1.15; 95% CI, 1.06–1.24; *P* = 0.0007), younger adults (OR, 1.25; 95% CI, 1.09–1.44; *P* = 0.001), and middle-aged adults (OR, 1.23; 95% CI, 1.05–1.44; *P* = 0.010), with significant linear trends in these groups (all P for trend < 0.010). No significant association was observed across UPF consumption quartiles in older adults (P for trend = 0.513).

**Table 3:** Association between ultra-processed food ratio and hyperuricemia stratified by age

Ultra-processed food ratio	Model 1	Model 2	Model 3
Total population			
Continuous	1.43 (1.28, 1.61)***	1.40 (1.25, 1.57)***	1.31 (1.15, 1.48)***
Quartiles			
Q1 (0-0.2)	Reference	Reference	Reference
Q2 (0.2–0.34)	1.01 (0.94, 1.08)	1.01 (0.94, 1.08)	1.02 (0.95, 1.10)
Q3 (0.34–0.51)	1.11 (1.03, 1.19)**	1.10 (1.03, 1.18)**	1.09 (1.01, 1.17)*
Q4 (0.51-1.00)	1.21 (1.13, 1.29)***	1.19 (1.11, 1.28)***	1.15 (1.06, 1.24)***
P for trend	< 0.001	< 0.001	< 0.001
Young adults (< 45 years)			
Continuous	1.64 (1.36, 1.97)***	1.51 (1.25, 1.82)***	1.35 (1.10, 1.66)**
Quartiles			
Q1 (0-0.2)	Reference	Reference	Reference
Q2 (0.2–0.34)	1.23 (1.07, 1.42)**	1.20 (1.05, 1.39)*	1.15 (0.99, 1.34)
Q3 (0.34–0.51)	1.31 (1.15, 1.50)***	1.27 (1.10, 1.45)***	1.21 (1.05, 1.40)**
Q4 (0.51-1.00)	1.42 (1.25, 1.62)***	1.35 (1.19, 1.54)***	1.25 (1.09, 1.44)**
P for trend	< 0.001	< 0.001	0.002
Middle-aged adults (45–59 years)			
Continuous	1.73 (1.38, 2.19)***	1.70 (1.34, 2.14)***	1.49 (1.15, 1.92)**
Quartiles			
Q1 (0-0.2)	Reference	Reference	Reference
Q2 (0.2–0.34)	1.04 (0.90, 1.20)	1.04 (0.89, 1.20)	1.03 (0.88, 1.21)
Q3 (0.34–0.51)	1.24 (1.07, 1.43)**	1.23 (1.06, 1.42)**	1.16 (0.99, 1.35)
Q4 (0.51-1.00)	1.33 (1.15, 1.53)***	1.31 (1.14, 1.52)***	1.23 (1.05, 1.44)*
P for trend	< 0.001	< 0.001	0.005
Older adults (≥ 60 years)			
Continuous	1.12 (0.93, 1.34)	1.13 (0.94, 1.36)	1.17 (0.95, 1.43)
Quartiles			
Q1 (0-0.2)	Reference	Reference	Reference
Q2 (0.2–0.34)	0.92 (0.84, 1.01)	0.92 (0.84, 1.01)	0.97 (0.87, 1.08)
Q3 (0.34–0.51)	0.97 (0.88, 1.07)	0.97 (0.87, 1.07)	0.98 (0.88, 1.10)
Q4 (0.51-1.00)	1.06 (0.95, 1.18)	1.07 (0.96, 1.19)	1.08 (0.96, 1.22)
P for trend	0.588	0.550	0.513

*Abbreviations: OR* Odds ratio, *CI* Confidence interval, *PIR* Poverty income ratio, *BMI* Body mass index, *METs* Metabolic equivalents, *eGFR* Estimated glomerular filtration rate

Model 1: Non-adjusted

Model 2: Adjusted for gender

Model 3: Adjusted for gender, race/ethnicity, PIR, education level, BMI, smoking, drinking, diabetes, hypertension, hyperlipidemia, METs/week, and eGFR

Data are presented as OR (95% CI). *P < 0.05, **P < 0.01, ***P < 0.001

Model diagnostic checks (Table S2) confirmed the validity of the fully adjusted logistic regression models. No evidence of multicollinearity was detected, with all generalized variance inflation factors (GVIF^(1/(2×Df))) < 1.17 (maximum 1.165 for eGFR). The Hosmer-Lemeshow goodness-of-fit test yielded χ² = 18.92 (df = 8, *p* = 0.015). The AUC was 0.756 (95% CI: 0.750–0.762), and the survey-weighted AUC was 0.752. E-values for the observed associations were 1.55 (lower 95% CI limit: 1.35) in the total population, 1.60 (lower limit: 1.28) in young adults, and 1.74 (lower limit: 1.35) in middle-aged adults.

Stratified analyses (Table 4) confirmed these findings, highlighting notable interactions that modify the UPF-hyperuricemia relationship, particularly in younger age groups. Among younger adults, significant interactions were observed for race/ethnicity (*P* = 0.010), alcohol consumption (*P* = 0.045), and diabetes (*P* = 0.050), indicating stronger associations in non-Hispanic White individuals, Mexican American individuals, current drinkers, and those with diabetes. In middle-aged adults, no significant interactions were noted (*P* > 0.050 for all), suggesting a consistent association across subgroups, consistent with the stronger ORs in Table 3. In older adults, although no significant overall association was observed (Table 3), significant interactions were observed for gender (*P* = 0.030), race/ethnicity (*P* = 0.020), and alcohol consumption (*P* = 0.020). Subgroup analyses revealed that the UPF-hyperuricemia association was significant among men (OR 1.47, 95% CI 1.10–1.95) but not women (OR 0.93, 95% CI 0.70–1.24), among non-Hispanic Black individuals (OR 1.81, 95% CI 1.21–2.72) but not other racial/ethnic groups, and among current drinkers (OR 1.40, 95% CI 1.06–1.84) but not never or former drinkers. These heterogeneous effects across subgroups likely explain the null overall association observed in older adults, as opposing or varying effect sizes may have attenuated the pooled estimate. These findings underscore that the UPF-hyperuricemia association is most pronounced and variable in individuals aged < 60 years, where demographic and clinical factors modify the strength of this association, whereas the absence of a significant association in older adults highlights the age-specific nature of this relationship.

**Table 4:** Stratified analyses of ultra-processed food ratio and hyperuricemia by age groups

Characteristics	Young adults (<45 years)		Middle-aged adults (45-59 years)		Older adults (≥60 years)	
	Adjusted ORs (95% CI)*	P-interaction	Adjusted ORs (95% CI)*	P-interaction	Adjusted ORs (95% CI)*	P-interaction
Gender		0.46		0.16		0.03
Male	1.28 (1.00, 1.64)		1.75 (1.24, 2.46)		1.47 (1.10, 1.95)	
Female	1.51 (1.06, 2.15)		1.22 (0.84, 1.78)		0.93 (0.70, 1.24)	
Race/ethnicity		0.01		0.31		0.02
Non-Hispanic White	1.66 (1.22, 2.27)		1.26 (0.85, 1.86)		0.87 (0.66, 1.16)	
Non-Hispanic Black	1.00 (0.63, 1.57)		2.20 (1.36, 3.55)		1.81 (1.21, 2.72)	
Mexican American	2.02 (1.24, 3.29)		1.25 (0.60, 2.59)		1.49 (0.84, 2.65)	
Others	0.82 (0.51, 1.30)		1.34 (0.73, 2.45)		1.28 (0.70, 2.33)	
PIR		0.40		0.75		0.98
Low	1.62 (1.15, 2.28)		1.65 (1.04, 2.62)		1.20 (0.83, 1.73)	
Medium	1.17 (0.84, 1.63)		1.55 (1.01, 2.37)		1.17 (0.86, 1.58)	
High	1.31 (0.89, 1.92)		1.31 (0.86, 1.99)		1.13 (0.76, 1.69)	
BMI (kg/m²)		0.58		0.24		0.13
≥25	1.47 (1.19, 1.83)		1.46 (1.12, 1.91)		1.09 (0.88, 1.37)	
<25	1.26 (0.77, 2.08)		2.27 (1.15, 4.48)		1.62 (1.02, 2.59)	
Smoking		0.17		0.09		0.48
Never	1.28 (0.98, 1.68)		1.26 (0.88, 1.81)		1.08 (0.80, 1.46)	
Former	0.99 (0.59, 1.66)		1.26 (0.76, 2.07)		1.15 (0.84, 1.58)	
Now	1.77 (1.21, 2.58)		2.41 (1.47, 3.97)		1.60 (0.91, 2.80)	
Drinking		<0.05		0.07		0.02
Never	1.19 (0.65, 2.18)		0.78 (0.38, 1.60)		0.63 (0.38, 1.03)	
Former	0.52 (0.27, 1.00)		1.16 (0.65, 2.08)		1.21 (0.83, 1.76)	
Current	1.55 (1.23, 1.94)		1.78 (1.32, 2.42)		1.40 (1.06, 1.84)	
Diabetes		0.05		0.68		0.06
No	1.27 (1.03, 1.58)		1.53 (1.15, 2.04)		1.35 (1.05, 1.74)	
Yes	2.62 (1.30, 5.30)		1.35 (0.79, 2.29)		0.90 (0.64, 1.27)	
Hypertension		0.35		0.33		0.11
No	1.27 (1.00, 1.62)		1.73 (1.17, 2.56)		0.85 (0.55, 1.32)	
Yes	1.58 (1.07, 2.31)		1.34 (0.97, 1.86)		1.27 (1.01, 1.60)	
Hyperlipidemia		0.60		0.10		0.57
No	1.24 (0.84, 1.82)		2.39 (1.29, 4.44)		1.35 (0.79, 2.29)	
Yes	1.40 (1.10, 1.77)		1.35 (1.03, 1.79)		1.14 (0.91, 1.42)	

Abbreviations: OR, odds ratio; CI, confidence interval; PIR, poverty income ratio; BMI, body mass index; METs, metabolic equivalents; eGFR, estimated glomerular filtration rate

Notes: *Each stratification adjusted for all the factors (gender, race/ethnicity, PIR, education level, BMI, smoking, drinking, diabetes, hypertension, hyperlipidemia, METs/week, and eGFR) except the stratification factor itself

**Fig. 2:**
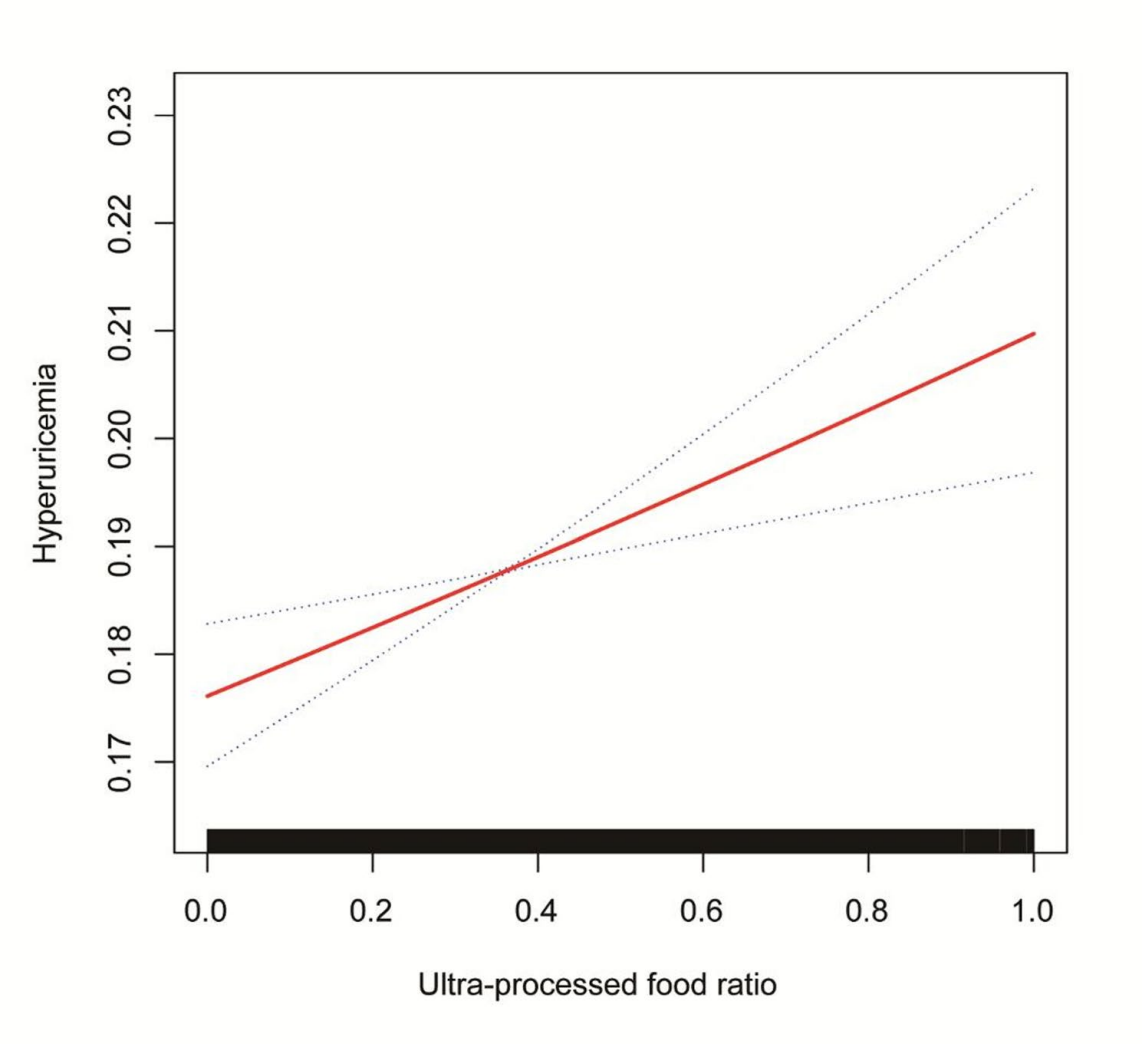
The association between ultra-processed food ratio and hyperuricemia. Notes: The solid red line represents the smooth curve from generalized additive model, and the dotted lines represent the 95% CIs. The black bars at the bottom indicate the distribution of ultra-processed food ratio values in the study population. Model was adjusted for gender, race/ethnicity, PIR, education level, BMI, smoking, drinking, diabetes, hypertension, hyperlipidemia, METs/week, and eGFR. Abbreviations: CI, confidence interval; PIR, poverty income ratio; BMI, body mass index; METs, metabolic equivalents; eGFR, estimated glomerular filtration rate

**Fig. 3:**
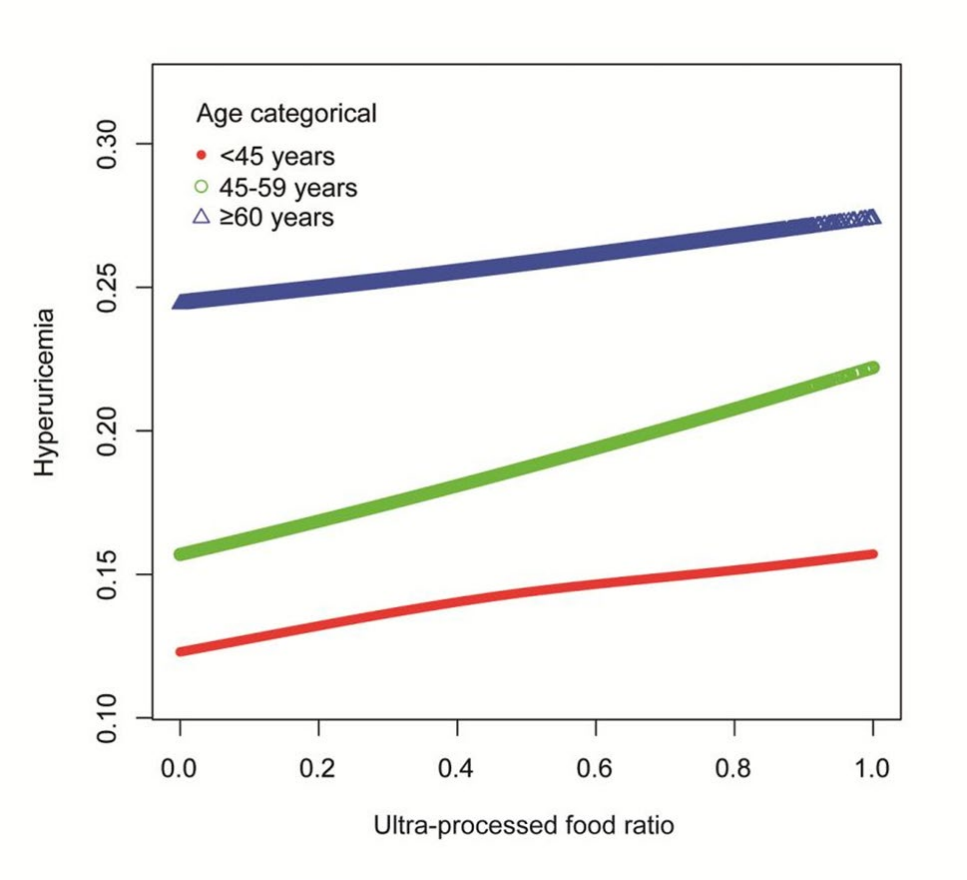
The association between ultra-processed food ratio and hyperuricemia among different ages. Notes: Red circles represent young adults (younger than 45 years), green circles represent middle-aged adults (45-59 years), and blue triangles represent older adults (60 years or older). Each generalized additive model and smooth curve fitting was adjusted for all factors, including gender, race/ethnicity, PIR, education level, BMI, smoking, drinking, diabetes, hypertension, hyperlipidemia, METs/week, and eGFR.Abbreviations: PIR, poverty income ratio; BMI, body mass index; METs, metabolic equivalents; eGFR, estimated glomerular filtration rate

To explore potential nonlinear relationships between the UPF consumption ratio and hyperuricemia, generalized additive models with smooth curve fitting were employed. In the total population (Fig. 2), after adjusting for gender, race/ethnicity, PIR, education level, BMI, smoking status, drinking status, diabetes, hypertension, hyperlipidemia, physical activity, and eGFR, the smooth curve demonstrated a monotonic positive association between UPF ratio and the predicted probability of hyperuricemia, with no evidence of a threshold effect or nonlinearity. The 95% CI (dashed lines) remained above the baseline throughout the UPF ratio range, indicating a consistent positive association. Age-stratified generalized additive model analyses (Fig. 3) revealed distinct curve shapes across groups: younger adults (< 45 years) and middle-aged adults (45–59 years) exhibited consistent upward trends, whereas the curve for older adults (≥ 60 years) remained relatively flat, suggesting that the dose-response relationship between UPF intake and hyperuricemia is attenuated in older populations.

**Fig. 4:**
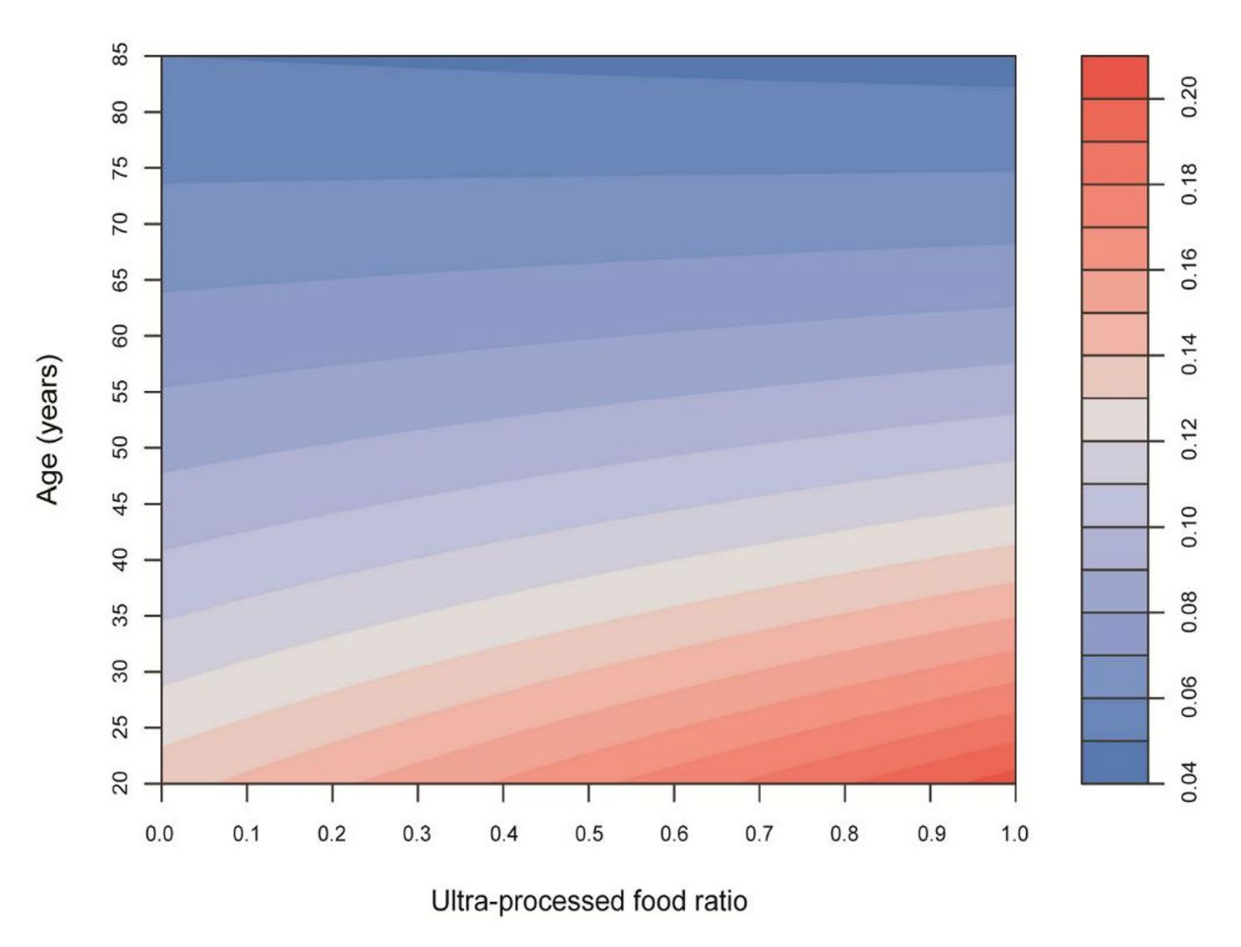
Heat map of potential interaction effect between ultra-processed food ratio and age on hyperuricemia. Notes: The heat map visualizes the estimated probability of hyperuricemia across different combinations of ultra-processed food ratio (x-axis, ranging from 0.0 to 1.0) and age (y-axis, ranging from 20 to 85 years) derived from logistic regression models. The color gradient represents probability values, with blue indicating lower probability (approximately 0.04) and red indicating higher probability (approximately 0.20). Models were adjusted for gender, race/ethnicity, PIR, education level, BMI, smoking, drinking, diabetes, hypertension, hyperlipidemia, METs/week, and eGFR. Abbreviations: PIR, poverty income ratio; BMI, body mass index; METs, metabolic equivalents; eGFR, estimated glomerular filtration rate

**Fig. 5:**
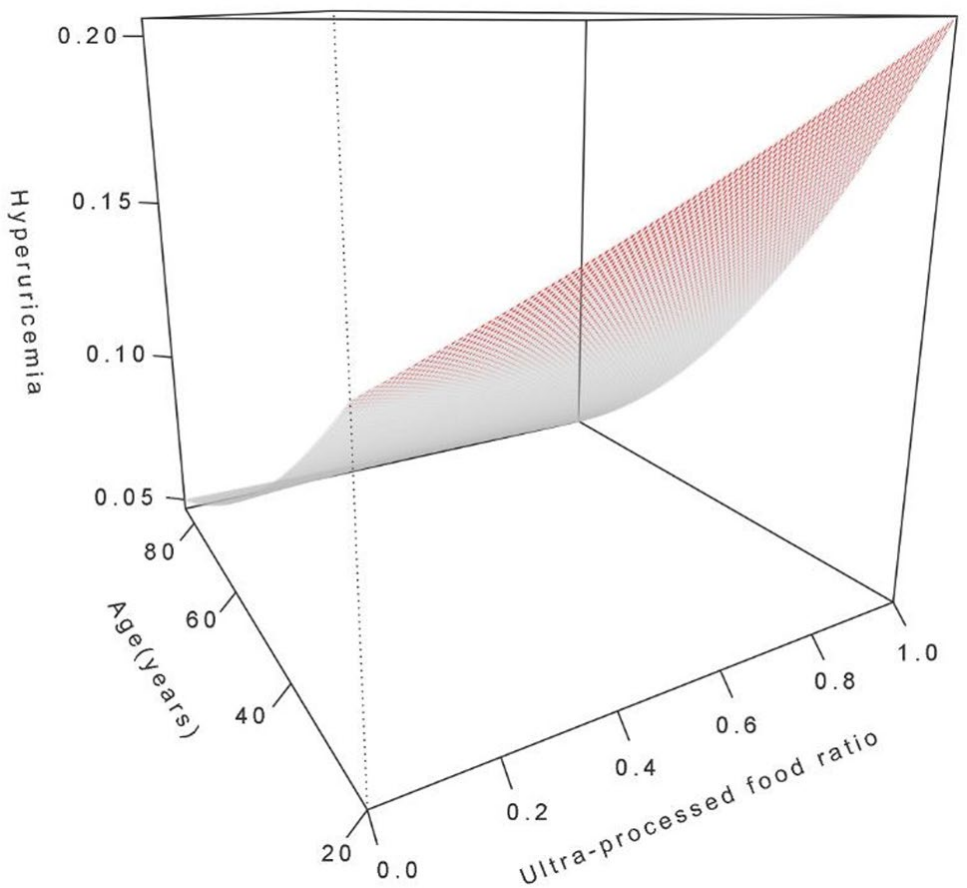
Three-dimensional diagram of potential interaction effect between ultra-processed food ratio and age on hyperuricemia. Notes: The three-dimensional surface plot displays the estimated probability of hyperuricemia (z-axis) as a function of ultra-processed food ratio (x-axis, ranging from 0.0 to 1.0) and age (y-axis, ranging from 20 to 85 years) derived from logistic regression models. The surface is color-coded from gray (lower probability) to red (higher probability). Models were adjusted for gender, race/ethnicity, PIR, education level, BMI, smoking, drinking, diabetes, hypertension, hyperlipidemia, METs/week, and eGFR.Abbreviations: PIR, poverty income ratio; BMI, body mass index; METs, metabolic equivalents; eGFR, estimated glomerular filtration rate

The interaction between the UPF consumption ratio and age on hyperuricemia was further examined through visual analyses. A heatmap (Fig. 4) illustrates the joint association of UPF consumption ratio and age with hyperuricemia probability, with darker red indicating a stronger association and darker blue indicating a weaker association. The heatmap reveals that higher UPF consumption ratios are associated with a greater probability of hyperuricemia at younger ages, where red bands are more prominent, whereas this association diminishes with increasing age, transitioning to blue in older adults. Complementing this, a three-dimensional plot (Fig. 5) depicts the interaction effect, showing that the probability of hyperuricemia associated with UPF intake decreases with advancing age, with a steeper increase in hyperuricemia probability at younger ages for higher UPF consumption ratios.

### Sensitivity analyses

Sensitivity analyses were conducted to assess the robustness of the primary findings to missing data handling. Missingness rates for covariates included in the regression models are detailed in Table S3, with physical activity (METs/week) showing the highest missingness (27.1%). Density plots confirmed plausible imputations, with imputed distributions closely overlapping observed distributions (Figure S1). In the complete case analysis (Table S4), the associations were largely consistent with the primary results, showing similar or slightly stronger effects in the total population and middle-aged adults, and comparable patterns of age modification across groups. In the sensitivity analysis additionally adjusting for urate-lowering therapy use (Table S5), results remained virtually unchanged, with ORs of 1.34 (95% CI 1.09–1.64) for young adults, 1.47 (95% CI 1.14–1.89) for middle-aged adults, and 1.16 (95% CI 0.95–1.42) for older adults, further supporting the robustness of the primary findings.

## Discussion

Using data from NHANES cycles spanning 1999–2018, this study identified a significant association between UPF consumption and hyperuricemia, particularly among individuals aged < 60 years. Logistic regression analyses showed that a higher UPF consumption ratio was associated with higher odds of hyperuricemia in the total population (OR, 1.31; 95% CI, 1.15–1.48; *P* < 0.001), with stronger associations in younger adults (< 45 years; OR, 1.35; 95% CI, 1.10–1.66; *P* = 0.004) and middle-aged adults (45–59 years; OR, 1.49; 95% CI, 1.15–1.92; *P* = 0.002) but not in older adults (≥ 60 years; OR, 1.17; 95% CI, 0.95–1.43; *P* = 0.138). These findings support our hypothesis that higher UPF intake is associated with higher odds of hyperuricemia, particularly in younger and middle-aged adults (< 60 years).

Our results build on prior research linking UPF consumption to uric acid metabolism disorders. Zhang et al. reported an association between UPF consumption and hyperuricemia risk in a prospective cohort, with a 16% increased risk among individuals in the highest UPF consumption quartile compared with the lowest [12]. Similarly, Fajardo et al. found that each 100 g/d increase in UPF intake was associated with higher serum uric acid levels, with increasing UPF intake raising hyperuricemia risk by 5.6% over approximately 4 years [11]. While these studies established a general relationship, our study uniquely identifies age as a critical effect modifier. Additionally, Zhang et al. found that UPF consumption was associated with higher odds of gout, particularly among genetically susceptible individuals, suggesting that individual factors, modify the relationship between diet and hyperuricemia and related conditions [10]. This pattern of age-related effect modification is consistent with recent findings showing that dietary pattern-health associations differ by age group, with weaker associations often observed in older adults [36], aligning with life-course epidemiology principles whereby dietary impacts vary across life stages [37]. 

The observed age-dependent associations may reflect both behavioral and physiological differences. Systematic reviews indicate that adults aged < 60 years consume approximately 50% more energy from UPFs than those aged ≥ 60 years [38], with younger adults, particularly men, consistently consuming more UPFs than older adults [39, 40]. Contributing factors include increased fast-food consumption during transitional life stages, reduced meal planning during stressful periods, and higher UPF consumption among younger adults experiencing socioeconomic disadvantage and lower educational attainment [41, 42]. Physiologically, age-related decline in renal function—which handles approximately 65%-75% of daily uric acid excretion—is strongly linked to elevated serum uric acid levels in older adults, potentially attenuating dietary effects [18, 43]. The stronger UPF-hyperuricemia association in middle-aged adults may be related to age-specific changes in adipose tissue distribution, as visceral fat accumulation is a stronger correlate of uric acid levels than subcutaneous fat [44]. Age-related differences in metabolic enzyme activity may also be relevant, potentially affecting how dietary components, such as fructose (prevalent in UPFs), relate to purine metabolism and uric acid excretion [45]. Genetic variants influencing uric acid metabolism also exhibit age-dependent expression patterns [46], suggesting complex gene-environment interactions across the lifespan.

Stratified analyses revealed key demographic and clinical modifiers of the UPF-hyperuricemia relationship. In younger adults, notable interactions were observed for race/ethnicity, alcohol consumption, and diabetes, suggesting that certain subgroups may exhibit stronger associations between UPF consumption and hyperuricemia. Racial/ethnic differences in uric acid handling [47], alcohol-related metabolic effects (adenosine triphosphate degradation and decreased uric acid excretion) [48, ], and the bidirectional relationship between hyperuricemia and insulin resistance likely explain these interactions [50–52]. In middle-aged adults, no significant interactions were observed, suggesting a consistent relationship across subgroups in this population. In older adults, notable interactions with gender, race/ethnicity, and alcohol consumption were identified, indicating that the UPF-hyperuricemia relationship varies across subgroups within this age group, even though the overall effect was null. Specifically, the significant positive associations observed among men (OR 1.47, 95% CI 1.10–1.95), non-Hispanic Black individuals (OR 1.81, 95% CI 1.21–2.72), and current drinkers (OR 1.40, 95% CI 1.06–1.84)—but not their counterparts—suggest that certain subpopulations of older adults may remain susceptible to the effects of UPF consumption on uric acid metabolism. The gender difference may be attributed to sex-specific variations in uric acid handling, as men typically have higher serum urate concentrations and lower fractional uric acid excretion than women [53]. The associations with race/ethnicity and alcohol consumption are consistent with the mechanisms described above [47–49]. The heterogeneous effects across these subgroups likely explain the null overall effect, as opposing or attenuated effects may have diluted the pooled estimate.

These findings have implications for clinical practice and public health strategies. Clinically, healthcare providers counseling patients on hyperuricemia management—particularly those under 60 years of age—may consider addressing UPF consumption as part of comprehensive dietary guidance. Specifically, reducing consumption of sugar-sweetened beverages, packaged snacks, and ready-to-eat meals may be particularly relevant for younger and middle-aged adults with concurrent diabetes or alcohol consumption, as our interaction analyses suggest stronger associations in these subgroups. Traditional dietary recommendations for hyperuricemia have focused on limiting purine-rich foods, but our results suggest that a broader approach emphasizing overall dietary patterns may be warranted. This reflects growing recognition that health effects of foods extend beyond nutrient composition to include processing-related additives and food matrix alterations. In older adults, where age-related renal decline predominates, dietary interventions targeting UPFs may have limited population-level effect, though certain subgroups (men, non-Hispanic Black individuals, current drinkers) may still benefit. These age-specific considerations suggest tailoring dietary counseling to individual age and risk profiles [54]. Beyond individual-level recommendations, population-level policies—such as those addressing food pricing, marketing, and labeling of UPFs—merit consideration.

This study has several strengths. To our knowledge, this is the first study to demonstrate that the UPF-hyperuricemia association varies significantly across age groups, with significant associations in adults aged < 60 years but not in older adults. The large, nationally representative sample from NHANES enhances generalizability, and comprehensive adjustment for potential confounders strengthens validity.

Several limitations should be acknowledged when interpreting these results. First, the cross-sectional design precludes causal inference, and reverse causation is a particular concern; for example, older adults with known hyperuricemia or gout may have intentionally modified their dietary habits on physician advice, potentially attenuating the observed UPF-hyperuricemia association in this age group [55]. This phenomenon, sometimes termed “healthy survivor effect” or survival bias, has been documented in nutritional epidemiology studies of older populations [56]. Longitudinal studies are needed to establish temporality. Second, despite comprehensive adjustment for confounders, residual confounding from unmeasured dietary patterns co-occurring with UPF consumption cannot be ruled out. E-value analysis indicated that an unmeasured confounder would need to be associated with both UPF consumption and hyperuricemia by a risk ratio of at least 1.35–1.74 to fully explain the observed associations in adults < 60 years, suggesting reasonably robust findings to unmeasured confounding of moderate strength. Third, dietary intake was assessed using 24-hour recalls, which are subject to limitations including recall bias, systematic underreporting, and day-to-day variation that may not fully capture habitual dietary patterns [57]. Despite use of the validated Automated Multiple-Pass Method, measurement error may have attenuated the true effects [58]. Fourth, limited sample sizes in some subgroups may have reduced statistical power for detecting interactions. Fifth, missing data on covariates, particularly physical activity (27.1% missingness), may have introduced bias. We employed multiple imputation under the missing at random assumption, and complete case sensitivity analysis yielded consistent results across age groups, supporting the robustness of our findings. Sixth, the classification of UPFs relied on the NOVA system, which, although widely accepted, may not fully capture all aspects of food processing relevant to uric acid metabolism.

Future research should employ prospective cohort designs to establish temporality and assess whether reducing UPF consumption is associated with decreased hyperuricemia incidence. Studies with objective dietary biomarkers could address the limitations of self-reported assessment. Mechanistic investigations are also needed to elucidate how UPF components influence uric acid metabolism across age groups.

## Conclusion

This study identified a significant age-dependent association between UPF consumption and hyperuricemia, with the strongest associations observed in middle-aged adults (45–59 years), followed by younger adults (< 45 years), while no significant association was observed among older adults (≥ 60 years). These findings suggest that age-specific dietary recommendations addressing UPF consumption may be relevant for hyperuricemia management, particularly in adults under 60 years of age.

## Supplementary Information

Supplementary Material 1

## Data Availability

Publicly available datasets were analyzed in this study. This data can be found here: NHANES website (https://www.cdc.gov/nchs/nhanes/index.htm).
