# Supplementary material for: Age modifies the relationship between ultra-processed food intake and hyperuricemia: findings from NHANES 1999–2018
Source: BMC Public Health. 2026 Jan 21;26:599. doi: 10.1186/s12889-026-26301-y (PMC12905914; doi:10.1186/s12889-026-26301-y)
Supplement: Supplementary file 1 — Supplementary Material 1 [file 12889_2026_26301_MOESM1_ESM.docx]

**Table S1. Categories defining Ultra-processed foods**

| **Category** | **Description** | **Examples** |
| --- | --- | --- |
| Sugary beverages | Carbonated drinks, energy drinks, fruit-flavored drinks with added sugars, sports drinks, and other sugar-sweetened beverages | Soft drinks; soda; cola; energy drinks; fruit-flavored drinks; sports drinks; sweetened coffee or tea beverages |
| Sweet or savory packaged snacks | Ready-to-eat products designed for direct consumption, typically high in salt, sugar, and/or fat | Potato chips; corn chips; extruded snacks; pretzels; flavored popcorn; crackers; puffed grain snacks |
| Confectionery | Products primarily made from sugar, often with added flavors, colors, and other ingredients | Candy bars; chocolate confections; gummy candies; hard candies; chewing gum; chocolate-covered snacks; marshmallows |
| Ice cream and frozen desserts | Sweet frozen products typically containing added sugars, flavors, emulsifiers, and stabilizers | Ice cream; frozen yogurt; sorbet; gelato; ice pops; frozen novelty desserts |
| Mass-produced packaged breads and buns | Commercially manufactured bread products containing additives such as emulsifiers, preservatives, and flavor enhancers | Sliced bread; hamburger buns; hot dog buns; sweet rolls; bagels; English muffins; wraps and flatbreads with additives |
| Cakes, cookies and pastries | Sweet baked goods containing refined flour, sugar, oils, and various additives | Packaged cookies; cakes; pies; pastries; donuts; muffins; sweet rolls; brownies |
| Breakfast cereals | Ready-to-eat or quick-preparation grain products, often with added sugar, salt, and various additives | Sweetened breakfast cereals; granola; cereal bars; instant oatmeal with added flavors and sweeteners |
| Pre-prepared and ready-to-heat products | Dishes prepared by industry that require minimal or no preparation besides heating | Frozen pizzas; ready meals; pasta dishes; nuggets; fish sticks; prepared sandwiches; instant soups; prepared meat dishes |
| Processed meat products | Meat products containing preservatives, flavor enhancers, and other additives beyond simple traditional preservation methods | Hot dogs; sausages; hamburger patties with additives; nuggets; reconstituted meat products; cured meats with nitrites/nitrates |
| Margarine and spreads | Spreadable products made from refined oils and containing emulsifiers, colors, and flavor enhancers | Margarine; vegetable fat spreads; flavored cream cheese spreads; chocolate spreads; sweet or savory spreads |
| Reconstituted food products | Foods made from powders, extracts, isolates, and other highly processed ingredients | Powdered soups; instant noodles; protein shakes; meal replacement drinks; infant formula; flavor enhancers |
| Artificially sweetened products | Foods and beverages containing non-nutritive sweeteners instead of sugar | Diet sodas; 'sugar-free' candies, desserts, and gum; low-calorie yogurts and ice creams with artificial sweeteners |

**Table S2. Additional diagnostic and sensitivity checks for the fully adjusted logistic regression model**

| **Diagnostic Check** | **Result** |
| --- | --- |
| Multicollinearity | All GVIF^(1/(2×Df)) < 1.17 (maximum 1.165 for eGFR) |
| Goodness-of-fit | Hosmer-Lemeshow test: χ² = 18.92, df = 8, p = 0.015 |
| Discrimination | AUC = 0.756 (95% CI: 0.750–0.762); survey-weighted AUC = 0.752 |
| E-value (total population) | Point estimate: 1.55; lower 95% CI limit: 1.35 |
| E-value (young adults, <45 y) | Point estimate: 1.60; lower 95% CI limit: 1.28 |
| E-value (middle-aged adults, 45–59 y) | Point estimate: 1.74; lower 95% CI limit: 1.35 |

**Abbreviations:** GVIF, generalized variance inflation factor; Df, degrees of freedom; AUC, area under the curve; CI, confidence interval; eGFR, estimated glomerular filtration rate.

**Table S3. Missingness rates for covariates included in the regression models**

| **Variables** | **N (total)** | **N (missing)** | **Missingness (%)** |
| --- | --- | --- | --- |
| Gender | 43,713 | 0 | 0.0 |
| Race/ethnicity | 43,713 | 0 | 0.0 |
| **PIR** | 43,713 | **3,587** | **8.2** |
| Education level | 43,713 | 43 | 0.1 |
| **BMI** | 43,713 | **612** | **1.4** |
| Smoking | 43,713 | 43 | 0.1 |
| **Drinking** | 43,713 | **3,105** | **7.1** |
| Diabetes | 43,713 | 0 | 0.0 |
| Hypertension | 43,713 | 0 | 0.0 |
| Hyperlipidemia | 43,713 | 0 | 0.0 |
| **METs/week** | 43,713 | **11,846** | **27.1** |
| eGFR | 43,713 | 0 | 0.0 |

**Abbreviations:** PIR, poverty income ratio; BMI, body mass index; METs, metabolic equivalents; eGFR, estimated glomerular filtration rate.

**Table S4.** **Complete case sensitivity analysis of the association between ultra-processed food ratio and hyperuricemia stratified by age (n=27366)**

| **Ultra-processed food ratio** | **Model 1** | **Model 2** | **Model 3** |
| --- | --- | --- | --- |
| **Total population** | 1.16 (1.00, 1.33)* | 1.12 (0.97, 1.29) | 1.66 (1.42, 1.94)*** |
| **Young adults (<45 years)** | 1.52 (1.22, 1.90)*** | 1.38 (1.11, 1.73)** | 1.27 (0.99, 1.61) |
| **Middle-aged adults (45-59 years)** | 1.93 (1.43, 2.60)*** | 1.89 (1.40, 2.54)*** | 1.80 (1.31, 2.47)*** |
| **Older adults (≥60 years)** | 1.28 (0.98, 1.66) | 1.29 (0.99, 1.68) | 1.40 (1.05, 1.87)* |

**Model 1:** Non-adjusted.

**Model 2:** Adjusted for gender.

**Model 3:** Adjusted for gender, race/ethnicity, PIR, education level, BMI, smoking, drinking, diabetes, hypertension, hyperlipidemia, METs/week, and eGFR.

Data are presented as OR (95% CI). *P < 0.05, **P < 0.01, ***P < 0.001.

**Abbreviations**: OR, odds ratio; CI, confidence interval; PIR, poverty income ratio; BMI, body mass index; METs, metabolic equivalents; eGFR, estimated glomerular filtration rate.

**Table S5. Sensitivity analysis additionally adjusting for urate-lowering therapy use**

| **Age group** | **OR (95% CI)** |
| --- | --- |
| **Total population** | 1.45 (1.29, 1.64)*** |
| **Young adults (<45 years)** | 1.34 (1.09, 1.64)** |
| **Middle-aged adults (45–59 years)** | 1.47 (1.14, 1.89)** |
| **Older adults (≥60 years)** | 1.16 (0.95, 1.42) |

**Notes:** Model adjusted for gender, race/ethnicity, PIR, education level, BMI, smoking, drinking, diabetes, hypertension, hyperlipidemia, METs/week, eGFR, and urate-lowering therapy.

Urate-lowering therapy was defined as self-reported use of allopurinol, febuxostat, or probenecid.

Data are presented as OR (95% CI). **P < 0.01, ***P < 0.001.

**Abbreviations:** OR, odds ratio; CI, confidence interval; PIR, poverty income ratio; BMI, body mass index; METs, metabolic equivalents; eGFR, estimated glomerular filtration rate.


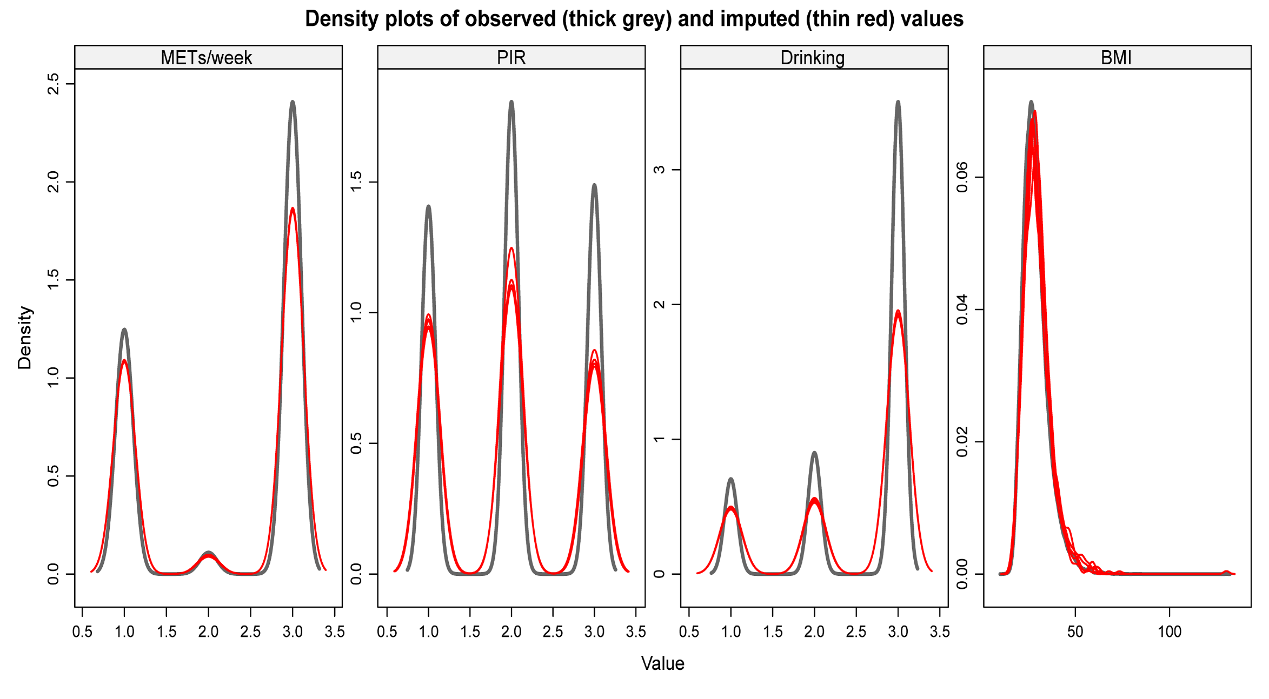


**Figure S1.** Density plots comparing observed and imputed values for covariates with missing data.

**Notes:** The plausibility of imputations was assessed using density plots comparing the distributions of observed (thick grey lines) and imputed (thin red lines) values for covariates with missing data. The four panels display the density distributions for METs/week (27.1% missing), PIR (8.2% missing), drinking status (7.1% missing), and BMI (1.4% missing). Similar distribution patterns between observed and imputed values suggest that the imputation model produced plausible values consistent with the observed data structure, supporting the validity of the multiple imputation approach under the missing at random assumption.

**Abbreviations:** METs, metabolic equivalents; PIR, poverty income ratio; BMI, body mass index.
